# Pathogenic interactions between *Helicobacter pylori* adhesion protein HopQ and human cell surface adhesion molecules CEACAMs in gastric epithelial cells

**DOI:** 10.22038/ijbms.2019.34237.81

**Published:** 2019-07

**Authors:** Ran Xia, Bo Zhang, Xinxin Wang, Qiuying Jia

**Affiliations:** 1 Geriatrics Department, the Affiliated Hospital to Changchun University of Chinese Medicine, Changchun, China; 2 Medical Record Room, the Affiliated Hospital to Changchun University of Chinese Medicine, Changchun, China

**Keywords:** CEACAM, Gastric epithelial cells, Helicobacter pylori, Interleukin 8, NF-kB

## Abstract

**Objective(s):**

The present paper aims to review the studies describing the interactions between HopQ and CEACAMs along with possible mechanisms responsible for pathogenicity of *Helicobacter pylori*.

**Materials and Methods:**

The literature was searched on “PubMed” using different key words including *Helicobacter pylori*, CEACAM and gastric.

**Results:**

HopQ is one of the outer membrane proteins of *H. pylori* and belongs to the family of adhesin proteins. In contrast to other adhesins, HopQ interacts with host cell surface molecules in a glycan independent manner. Human CEACAMs are the cell surface adhesion molecules mainly present on the epithelial cells, endothelial cells and leukocytes. The overexpression of these molecules may contribute to cancer progression and relapse. Recent studies have shown that HopQ may interact with human CEACAMs, particularly CEACAM1, CEACAM3, CEACAM5 and CEACAM6, but not CEACAM8. HopQ interacts with GFCC’C” interaction surface of IgV domain of N- terminal region of CEACAM1. Moreover, binding of HopQ to CEACAM1 prevent its trans-dimerization and stabilizes it in monomeric form. *H. pylori* may use these HopQ-CEACAM interactions to transfer its CagA oncoprotein into host gastric epithelial cells, which is followed by its phosphorylation and release of interleukin-8. HopQ-CEACAM interactions may also utilize T4SS, instead of CagA, to activate NF-κB signaling and trigger inflammation.

**Conclusion:**

HopQ of *H. pylori* may interact with CEACAMs of the human gastric cells to induce the development of gastric ulcers and cancers by transferring CagA oncoprotein or inducing activation of T4SS to initiate and maintain inflammatory reactions.

## Introduction

*Helicobacter pylori *is one of the oldest known bacterium carried by humans, more than 100,000 years, and humans along with primates, are the only known hosts of this bacterium. The bacterium resides and colonizes the stomach of the humans. Another important fact is that infection of *H. pylori* is very common and about 50% of persons of whole world are infected with this bacterium (1, 2). Different factors responsible for its unique adaptability in human stomach include its ability to tolerate low gastric pH (3), as well as its exceptional ability to escape from innate and adaptive host immune responses (4). Infection with this bacterium is associated with diverse manifestations ranging from asymptomatic gastritis to gastric and duodenal ulcers, and mucosa associated lymphoid tissue (MALT) lymphoma, gastric lymphoma and gastric adenocarcinoma (5-8). The clinical studies have shown that degree of *H. pylori* infection is directly correlated with severity of inflammation and activation of immune response. There is an increase in numbers of CD4+ and inflammatory cytokines in *H. pylori* infected persons in comparison to non-infected persons and the number is much higher in gastric ulceration than gastritis (9).

The outer membrane proteins present on the surface of *H. pylori *play a critical role in binding to human gastric epithelial cells (10). The high prevalence of *H. pylori* infection and its unique adaptability in human gastric epithelium is also attributed to rapid changes in expression of outer membrane proteins. The subtle changes in these proteins confer adaptability to *H. pylori* and facilitate persistent colonization of gastric epithelium (11). Helicobacter outer membrane proteins (Hop) constitute a family of adhesin proteins, which bind to glycans and proteins components of the host cells (12). It has been reported that HopQ control the adherence *H. pylori* to the human gastric epithelial cells. Amongst different Hop, HopQ is an important member and is found to interact with carcinoembryonic antigen-related cell adhesion molecules (CEACAMs) present on the human gastric epithelial cells (13). 

CEACAMs are the members of immunoglobulin superfamily and carcinoembryonic antigen (CEA) family. These are cell surface glycoproteins and act as cell adhesion proteins. These are widespread in the body and located on the epithelial cells, endothelial cells, WBCs and myeloid cells (14-16). Being adhesion molecules, they make contact with molecules of same type (homotypic binding) as well as of different types (heterotypic binding) and tend to maintain the tissue architecture (17). CEACAMs serve to control wide range of biological activities including cellular proliferation, cell signaling and cell differentiation. Studies have shown their critical role in regulating cell communication in different types of cancer (18). These molecules primarily act as tumor suppressants and inhibit development of cancer (19). Nevertheless, overexpression of these molecules leads to cancer development by producing aberrant cell differentiation, inhibiting apoptosis and promoting cell survival (17). Furthermore, high expression of these molecules is also positively correlated with high rate of cancer recurrence (20). 

Recent studies have focused on the interactions between HopQ and human CEACAMs, particularly, CEACAM1, CEACAM3, CEACAM5 and CEACAM6. In contrast, CEACAM8 was not able to exhibit appreciable binding with HopQ (21-24). The increased expression of CEACAMs has been documented on the gastric epithelial cells in patients suffering from gastric cancer in comparison to healthy, non-infected persons (22) suggesting the key role of CEACAM in cancer development and progression. 

Since the interactions between HopQ of *H. pylori *and CEACAMs of the host cells is a relatively new concept, therefore, the goal of the present paper is to review the studies describing the interactions between HopQ and CEACAMs along with possible mechanisms responsible for pathogenicity of* H. pylori*. It may be proposed that these interactions may account for the development of different types of gastric cancers associated with persistent *H. pylori* infection. Accordingly, the present review discusses the pathogenic interactions between HopQ and human CEACAM on which *H. pylori* may be dependent for producing gastritis, ulceration and cancer. 

***HopQ as one of the types of Hop proteins***

The genome of *H. pylori* has thirty Hop genes and Hop-related (Hor) gene families, which function to encode a family of adhesin proteins, collectively termed as Helicobacter outer membrane proteins (Hop) (12, 25). These bacterial adhesin Hop interact with glycans as well as with proteins present on the surface of the host cells. BabA and SabA are the examples of glycan recognition bacterial adhesins. BabA is found to interact with blood group antigen fucosylated-Lewis B (Le^B^) antigens. On the other hand, SabA is found to interact with blood group antigen sialylated Lewis X (sLe^X^). These binding interactions are important for the persistence of *H. pylori *in the stomach and responsible for persistent infection and inflammation (26, 27). The other examples of Hop proteins for which host cell receptors are not identified include HopC, HopB, HopH and HopZ. Some Hop proteins are reported to act as porins and these include HopV, HopW, HopX, and HopY (28).

HopQ is an important member of Hop family and it is found to interact with CEACAMs present on the human (host) cells (21, 22). There is a very little structural similarity between HopQ and BabA (about thirty five percent) and HopQ and SabA (about twenty three percent). However, the important point is that the similarity is found in the extracellular domains, which is primarily responsible for binding. Despite the extensive glycosylation in the extracellular loops of HopQ, it is interesting to note that the binding of HopQ with CEACAM1 is glycan independent. This property differentiates HopQ from other Hop adhesins, which primarily interact in a glycan-dependent manner (21, 22). 

There are two types of HopQ i.e., type I and type II and these two types have seventy percent of structural similarity (29). It has been documented that type I HopQ is present in the strains of *H. pylori* that possess ‘cag pathogenicity island’ (PAI) gene. This gene is responsible for the formation of pathogenic CagA protein (oncoprotein) and type IV secretion system (T4SS). This secretion system plays an important role in transferring pathogenic protein CagA from *H. pylori* to human gastric epithelial cells (30). Accordingly, *H. pylori* strains with type I HopQ variants are more pathogenic and trigger greater degree of gastric inflammation and atrophy (31). Moreover, there have been studies showing co-existence of type I Hop Q and virulence markers such as type S1 *vac A*. On the contrary, type II HopQ is present in the strains of *H. pylori* that do not possess ‘cag pathogenicity island’ gene. Moreover in these strains, type S1 *vac A* virulence gene is absent. Another difference is that type I HopQ is found in strains present in Eastern countries, while type II HopQ is present in strains isolated from Western countries (31). Therefore, it may be summarized that type I HopQ is most prevalent in Eastern type strains with cagA-positive and S1 *vac A *genotypes, while HopQ type II is found more commonly in Western strains with cagA-negative, *vac A* S2 genotype.

***Carcinoembryonic antigen-related cell adhesion molecules (CEACAMs)***

CEACAMs refer to carcinoembryonic antigen-related cell adhesion molecules and in human beings, 12 members constitute a complete CEACAM family. The individual protein members of this family have common structures. Structurally, there is Ig variable (IgV) domain at its N-terminal and it is proposed to be the major site for different types of interactions. There are 3 N-linked glycosylation sites at the N-terminal IgV domain of CEACAM1 (16). These adhesion molecules are widely distributed in the body. Nevertheless, each member has tissue specific distribution. CEACAM1 are mainly expressed on the WBCs, endothelial and epithelial cells (32, 33); CEACAM5, CEACAM7 and CEACAM6 are present on the epithelial cells; while CEACAM3 and CEACAM8 are located on the granulocytes (34). CEACAM6 are localized on the epithelial cells of spleen, lymph nodes, lung, esophagus and stomach (14, 15). It is mainly secreted as soluble receptor and there is a large similarity between CEACAM 1 and CEACAM6 in terms of binding interactions with different epitopes. CEACAM3, localized on the leukocytes, serve as innate immune receptors and help in promoting phagocytosis (34, 35). CEACAM1 are located on the apical as well as the basolateral side of the epithelial cells (36).

Amongst these, CEACAM1 has received much attention and its twelve variants have been documented in humans. The major variants include CEACAM1-4L, CEACAM1-3L, CEACAM1-4S, and CEACAM1-3S (37-39). There is an important role of receptor bound cytoplasmic ITIM domain, which is acted upon by Src tyrosine kinases to induce phosphorylation of ITIM domain. It has been shown that the phosphorylated form of ITIM domain is able to control downstream signaling events by modifying tyrosine phosphatases such as SHP-1 and SHP-2 (40, 41).

**Figure 1:**
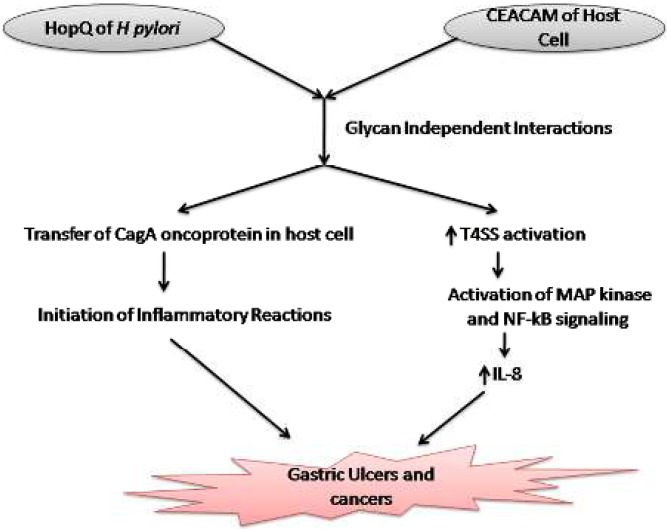
Schematic representation of glycan independent interactions of HopQ of *Helicobacter pylori* and CEACAMs of human epithelial cells leading to transfer of CagA oncoproteins and activation of T4SS, which is followed by activation of inflammatory reactions to induce gastric ulcers and cancers

***Interactions between HoP and CEACAMs***

It has been suggested that *H. pylori *may target the human CEACAMs to gain entry inside the human cells and CEACAMs serve as striking targets for bacteria because these protein molecules are widely expressed on the surface of gastric epithelial cells. Moreover, bacteria may also target WBC localized CEACAM1 to interfere and attenuate the host immune response, which is essential for the survival of bacterium inside human stomach (37). Using CEACAM expressing cell lines (*in vitro* studies), it has been shown that HopQ exhibit very affinity for different CEACAMs (K_D_ range = 23 to 268 nM) and binds to CEACAM1, CEACAM3, CEACAM5, and CEACAM6. However, HopQ did not show affinity with CEACAM8 and it was unable to bind with this adhesion molecule (21, 22). The binding of HopQ was localized to the IgV domain of the N-terminal region and binding was independent of CEACAM glycosylation. Moreover, it is reported that CEACAM3 has affinity for HopQ, which is about 2 times lesser than with CEACAM1 (24).

***Binding characteristics of HopQ with CEMEM1***

A very recent study has shown that type I HopQ binds to N-terminal domain of human CEACAM1 (C1ND) according to the ‘induced fit model’. The interaction site of HopQ consists of two conserved cysteine pairs, also termed as cysteine-clasped loop 1 and 2 (CL1 and 2), which group at the top surface to interact with the C1ND. On the other hand, Ig variable (Igv) domain of C1ND constitutes an interacting domain, in which GFCC’C” serves as a principal interaction surface. The cysteine loops of HopQ interact with GFCC’C” interaction surface of Ig variable domain of C1ND by hydrophobic as well as hydrogen bonding (23). Moreover, the same study showed that type II HopQ also binds to the same site of C1ND. However, the significant differences include lesser number of hydrogen bonds in the type II HopQ-C1ND complex in comparison to type I HopQ-C1ND. Interestingly, type II HopQ exhibited 6 times more affinity for C1ND (K_D_=69 nM) in comparison to type I HopQ (K_D_=417 nM). It has also been shown that the binding of type I HopQ with C1ND is enthalphy driven, whereas binding of type II HopQ is entropically driven (23).

***HopQ promotes monomeric form of CEACAM1***

CEACAM1 molecules can participate in *cis-* as well as *trans-*dimerization interactions. *Cis-* interactions involve interactions on a single-cell surface, whereas *trans-* interactions involve interactions across cell junctions. These interactions are very important in signal transduction from outside of a cell to the interior of cell (37). Studies have shown that *cis-*oligomerization utilizes ‘ABED interaction surface’ of N-terminal domain of CEACAM1. On the other hand, *trans-* dimerization utilizes ‘GFCC’C’’ interaction surface’ of N-terminal domain of CEACAM1. It is also interesting to note that same GFCC’C’’ interaction surface of N-terminal domain of CEACAM1 is also involved in interaction with HopQ (42). Therefore, it is possible to hypothesize that HopQ may compete for binding with the GFCC’C’’ interaction surface and prevent *trans-* dimerization of CEACAM1. This hypothesizes was experimentally verified by Moonens and co-workers who demonstrated that HopQ binds with CEACAM1 to prevent its *trans-* dimerization and stabilizes in monomeric form (23).

It is demonstrated that C1ND primarily exists in *trans-* dimer form in the solution. Indeed, there is equilibrium between the dimeric and monomeric forms of *trans-* C1ND. HopQ interacts with GFCC’C’’ interaction surface of C1ND to favor the monomeric form and disrupt the dimeric form of *trans-* C1ND. Indeed, interaction of HopQ with C1ND prevent the formation of the C-dimer, which otherwise is very crucial for mediating *trans-* as well as *cis-* interactions. (23). It may be possible to speculate that HopQ mediated disruption of the dimer form of CEACAM may be lead to formation of highly flexible monomeric form of CEACAM, which may trigger intracellular signaling pathway responsible for immune suppression and inflammation. An earlier study of Klaile *et al.* has shown that the monomeric form of CEACAM1-4 is very flexible and has extended shapes (42), which is essential for transducing intracellular signaling cascade. 

***HopQ-CEACAM1 interactions promote passage of Cga A oncoprotein***

Scientists have shown that *H. pylori* use HopQ-CEACAM interactions to transfer its Cag A oncoprotein into host gastric epithelial cells. Moreover, it is also followed by phosphorylation of Cag A protein, initiation of inflammatory reactions and release of pro-inflammatory cytokines including interleukin-8 (21, 22, 24). Structurally, CAECAM1 have ITIM (immunoreceptor tyrosine-based inhibition motif) domain, which is present in the cytoplasm and attached to membranous parts of adhesion molecules. This motif has a significant role in signal transduction in CEACAM1-mediated functions. However, the study of Moonens *et al.* reported that there is no significant role of CEACAM1’s cytoplasmic ITIM domain in HopQ-mediated transfer and phosphorylation of bacterial Cag A oncoprotein in the host (human) cells (23).

***HopQ-CAMCAM1 interactions activate NF-kB and MAP signaling to increase IL-8***

It has been shown that HopQ-CAMCAM1 interactions may increase the production of inflammatory mediator, IL-8 by activating NF-kB and mitogen activated protein (MAP) signaling. In a very recent study, Feige *et al.* reported that HopQ is capable of activating NF-κB in CEACAM expressing cell lines including AGS and NCI-N87 cells. In contrast, HopQ failed to modulate NF-κB in cell lines devoid of CEACAM such as HeLa cells. Moreover, the authors suggested that HopQ does not directly activate NF-κB. Instead, HopQ-induced increase in NF-κB activity was indirectly dependent on increased functionality of another virulence factor i.e., T4SS and independent of CagA (43). Earlier studies have also reported that *H. pylori *is capable of activating NF-κB, which is dependent on activation of T4SS, but independent of Cag A oncoprotein (44, 45). Belogolova *et al.* reported that HopQ activates NF-κB as well as MAP kinases indirectly through facilitation of T4SS. The authors showed that specific deletion of HopQ genes significantly abolished T4SS-dependent activation of NF-κB, MAP kinase signaling and release of inflammatory cytokines 8 (IL-8)(46). Studies have shown that activation of MAP kinase and NF-κB is involved in activating IL-8 genes. Moreover, *H. pylori *have been shown to increase the production of IL-8 in the gastric epithelial cells through NF-κB. Along with it, Hop is also shown to increase IL-8 production in host cells (47,48). Therefore, it may be proposed that HopQ interact with human CEACAM to facilitate T4SS and increase the production of IL-8 through activation of NF-κB and MAP kinase signaling cascade.

## Discussion

HopQ is an important member of a family of adhesin proteins of *H. pylori*, which bind to glycans and proteins components of the host cells (12). Recent studies have shown their interaction with CEACAMs present on the human gastric epithelial cells (21-24). CEACAMs are the cell surface adhesion molecules, which are mainly located on the epithelial cells, endothelial cells, WBCs and myeloid cells (14-16). At basal level, these serve to these molecules maintain the tissue architecture and act as tumor suppressants (19). However, overexpression of these molecules may participate in angiogenesis, cancer progression and cancer relapse (17, 20). An increase in expression of CEACAMs has been documented on the gastric epithelial cells in patients suffering from gastritis or gastric cancer in comparison to healthy, no-infected persons (22). Therefore, HopQ-CEACAM interactions may possibly contribute in the development of gastric cancers due to persistent *H. pylori* infection. 

The cell lines-based *in vitro* studies have shown the significant interactions between *H. pylori *adhesin HopQ and human CECAM, particularly CEACAM1, CEACAM3, CEACAM5, and CEACAM6, but not with CEACAM8 (21-24). HopQ interacted with the GFCC’C” interaction surface of IgV domain of the N-terminal region of CEACAM1 in a glycan independent manner. Moreover, binding of HopQ to CEACAM1 prevent its *trans-* dimerization and stabilizes it in monomeric form (23), which is more flexible form (42) and it may trigger intracellular signaling pathway in the host cell responsible for inflammation. Scientists reported that *H. pylori *may utilize these HopQ-CEACAM interactions to transfer its CagA oncoprotein into host gastric epithelial cells. The transfer of bacterial CagA in the host cell is followed by its phosphorylation and initiation of inflammatory reactions including release of pro-inflammatory interleukin-8 (21, 22, 24). CEACAM1’s cytoplasmic ITIM domain does not participate in transfer and phosphorylation of bacterial CagA oncoprotein in the host cells (23). The steps subsequent to CagA transfer and its phosphorylation are not clearly elucidated. The precise role of CagA in production of IL-8 is not clear as the study of Feige *et al.* reported that HopQ-CEACAM interactions utilize T4SS, instead of CagA, to activate NF-κB signaling (43). Other studies also document that activation of NF-κB during *H. pylori *infection is dependent on T4SS activation and independent of CagA oncoprotein (Figure 1) (44, 45). Therefore, signaling cascade triggered in host human cells following HopQ-CEACAM interactions are still not clear.

***Future directions***

Future research studies should be directed to understand the functional relevance of bacterial HopQ and human CEACAM interactions with respect to development of gastritis, ulcers or cancers. Most of these studies documenting these interactions are *in vitro* studies and are based on results obtained from cell lines. There is a need of *in vivo *studies to fully elucidate the pathogenic role of these interactions in development of disease particularly gastric cancer, considering the reported role of these interactions in transferring CagA oncoprotein in host cells. Moreover, studies are also required to elucidate the signaling cascade triggered in host cells following HopQ-CEACAM interactions.

## Conclusion

HopQ is an important protein of *H. pylori* and it may interact with CEACAMs of the human cells to induce the development of gastric ulcers and cancers. *H. pylori* may be dependent on these interactions to transfer CagA oncoprotein or induce activation of T4SS to initiate and maintain inflammatory reactions to induce gastric diseases including gastric cancer. Therefore, successful development of new drugs targeting to inhibit HopQ-CEACAM interactions may possibly contribute in effective management of *H. pylori*-induced gastric diseases. 

## Conflict of Interest

The authors declare no conflict of interest.
